# Improving the Enrichment of Submicron-Sized Particles by Size Decreasing of Cruciform Cross-Sectional Microchannel in Viscoelastic Microfluidics

**DOI:** 10.3390/bios15060370

**Published:** 2025-06-09

**Authors:** Jaekyeong Jang, Eunjin Kim, Sungdong Kim, Ok-Chan Jeong, Sangwook Lee, Younghak Cho

**Affiliations:** 1 Department of Mechanical Design and Robot Engineering, Seoul National University of Science and Technology, Seoul 01811, Republic of Korea; jjangy5720@naver.com (J.J.); sdkim@seoultech.ac.kr (S.K.); 2 PCL Inc., Seoul 08510, Republic of Korea; kitty97@oasis.inje.ac.kr; 3 Department of Biomedical Engineering, Inje University, Gimhae-si 50834, Republic of Korea; memsoku@inje.ac.kr; 4 Department of Mechanical System Design Engineering, Seoul National University of Science and Technology, Seoul 01811, Republic of Korea; 5 mCureX, Seoul 05854, Republic of Korea

**Keywords:** cruciform microchannel, viscoelastic fluid, reflex angle, submicron-sized particle, focusing and enrichment

## Abstract

The manipulation of cells and bioparticles has garnered significant interest in the field of viscoelastic microfluidics, particularly regarding its capacity for single-stream focusing within a three-dimensional and simple microchannel structure. The inherent simplicity of this method enables the effective manipulation of particles, facilitating the separation and focusing of various cell types, including blood cells, circulating tumor cells (CTCs), and microalgae. However, the viscoelastic nature of the particles imposes limitations in the handling of submicron-sized particles, due to a significant decrease in the viscoelastic force acting on the particle. In this study, we propose a microfluidic device featuring a cruciform cross-sectional microchannel with 45 µm and 45 µm of its vertical and horizontal size, respectively. The cruciform microchannel, which has a 270° reflex angle on four corners, can increase the viscoelastic force on the particles, allowing the device to focus submicron-sized particles down to 180 nm in a single-stream manner. It is important to note that the single-stream formation was maintained, while the channel width at the outlet region was drastically increased, allowing for the enrichment of submicron-sized particles. For biological feasibility, the proposed device also demonstrates the single-stream focusing on biological particles such as bacteria. The presented microfluidic device would have great potential for the focusing and enrichment of nanoparticles including bacteria in a highly robust manner, expecting its use in the various fields such as diverse biological analysis and biomedical research.

## 1. Introduction

Focusing submicron-sized particles in a single stream is of great importance for high throughput and accurate analysis of bioparticles such as bacteria, viruses, and exosomes in flow cytometry and fluorescence-activated cell-sorting (FACS) [1,2,3,4,5]. Microfluidics has shown significant potential for submicron particle focusing via robust and low-cost lab-on-a-chip platforms. A number of reviews have been presented to facilitate the understanding of the development process, recent advances, and future prospects of microfluidic methods for focusing and manipulating submicron/nanoscale particles [6,7]. According to their basic mechanisms, the methods can be divided into two categories: active methods, which require an external field such as electrical, acoustic or optical forces to drive the particle movement (e.g., electrophoresis, dielectrophoresis, acoustophoresis, etc.), and passive methods, which rely solely on the intrinsic property of the fluids within the channel to generate forces (e.g., filtration, deterministic lateral displacement (DLD), inertial/viscoelastic microfluidics) [6,7,8].

Recently, the field of viscoelastic microfluidics has garnered significant attention due to its ability to manipulate submicron-sized particles within a minute sample volume, obviating the need for labeling or the application of external force fields. This development signifies a substantial advancement in technology, allowing for precise and effective control over submicron/nano-sized particles [9,10]. The manipulation of particles within a viscoelastic fluid is found to be significantly influenced by several parameters, including the size of the particles, the flow rate, the cross-sectional shape and length of the channel, and the concentration of the polymer solution. Numerous studies have demonstrated the efficacy of manipulating submicron/nano-sized particles within rectangular microchannels by employing the Dean flow in spiral channels or by increasing the polymer concentration of the fluid solution [11,12,13]. Liu et al. demonstrated the sheathless manipulation of a variety of nanoparticles such as synthetic nanoparticles, λ-DNA molecules, and DNA origamis in a viscoelastic fluid using a double spiral microchannel [11]. Asghari et al. presented sheathless oscillatory viscoelastic microfluidics as a method for focusing and separating particles that ranged in size from 20 nm up to several micrometers [12]. Owing to the shrinkage of channel size, it was possible to focus nanoparticles at specific locations based on their size. However, they suffered from the low throughput of sample treatment and the channel clogging due to the small channel size (channel height of 4 µm and 0.7 µm). A microchannel with a gradually contracted cross-section and high-aspect-ratio was fabricated and tested to enrich the submicron/nano-sized particles in a viscoelastic fluid [13]. The fabricated microchannel significantly changed the distribution of the elastic lift force and increased the dominant driving forces, which induced the efficient enrichment of the target particles in a short channel length over a wide range of flow rates and particle concentration. However, the focusing and enrichment of less than 500 nm particles with low blockage ratio (*β* < 0.07, see the Section 2.2) was not presented.

In our preceding study [14], we effectively engineered a microchannel with a cruciform cross-section, exhibiting four corners with reflex angles of 270°. We conducted particle focusing experiments at various flow rates and particle sizes under viscoelastic fluid flow. Our investigation focused on the impact of these parameters on the position of the particles. A comparison of microchannels with square and cruciform cross-sections revealed that the latter could more efficiently focus micron-sized particles to a single stream without any shear thinning. Here, as an improvement in particle focusing down to the submicrometer range, we proposed the cruciform microchannel with a 67% smaller cross-section than that reported previously. Focusing and enriching performance was evaluated under various experimental conditions including particle size, flow rate, and PEO concentration at different molecular weights. Up to 180 nm particles could be focused and enriched in the center of the microchannel at a relatively high flow rate under viscoelastic fluid flow. Moreover, we tested focusing and enriching performance using bacteria cells to demonstrate the biological relevance of the device. The fabricated microfluidic device with the cruciform microchannel could align submicron-sized particles within a narrow flow stream in a highly robust manner and enrich the biological samples such as bacteria in the center outlet. Although the fabrication process of a cruciform cross-sectional microchannel is comparably more complicated than a square one, it revealed that the latter could more efficiently focus submicron-sized particles to a single stream without any shear thinning. Thus, we proposed downsized cruciform cross-sectional to improve particle focusing down to the submicrometer range. This flow-through focusing platform has the potential to address a wide range of applications including point-of-care diagnostics, bioassays, rare sample processing, and submicron/nano-flow cytometry [15].

## 2. Materials and Methods

### 2.1. Device Fabrication and Sample Preparation

**Figure 1:**
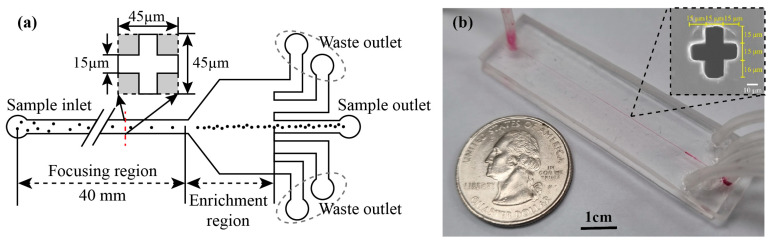
(**a**) Schematic view of microfluidic device and its cruciform cross-section of microchannel; (**b**) optical photo of fabricated microfluidic device and its SEM image of the microchannel.

As shown in Figure 1, the cruciform microchannel was fabricated using the same way as in our previous report, which was based on SU-8 lithography and PDMS molding followed by self-alignment between two PDMS molds [14]. The whole chip is composed of a single inlet and the five outlets; the one in the middle is the sample outlet and the others are waste. The microchannel in the chip is divided into two parts: one to focus particles on the center of the microchannel and the other to enrich the particles; the focused particles are flown into the sample outlet and the other solution comes to the waste outlets. The upper inset in Figure 1a presents the geometric detail of the cruciform cross-section, where one side of the cruciform microchannel is 15 µm and its total width and height is 45 µm. Therefore, its hydraulic diameter (*D_h_*) is calculated as 25 µm. Figure 1b presents the optical photo of fabricated microfluidic device and its cross-sectional scanning electron microscope (SEM) image of cruciform microchannel, which is formed by the perfect self-alignment between two PDMS molds. As a comparison, we also fabricated a square microchannel with a geometrical cross-section of 25 μm × 25 μm, where its hydraulic diameter (*D_h_*) is similar to that of cruciform channel. Tables S1 and S2 show the pressure drop and channel deformation in the square and cruciform microchannels depending on the flow rate [16].

In this work, a polyethylene oxide (PEO) solution was used as a viscoelastic fluid, with concentrations ranging from 0.05 to 0.5 wt%. To make the PEO solution, PEO (~0.6 and ~2 MDa, Sigma-Aldrich, Burlington, MA, USA) was dissolved in DI (Deionized) water. Their rheological properties are shown in Table S3 [11]. Fluorescent polystyrene (PS) particles (Thermo Scientific Inc., Waltham, MA, USA) with 510 nm (1.5 × 10^7^ particles/mL), 250 nm (1.2 × 10^8^ particles/mL), 180 nm (2.3 × 10^8^ particles/mL), and 100 nm (3.6 × 10^8^ particles/mL) diameters were placed in the PEO solution. To prevent particle aggregation during the experiments, Tween 20 (Sigma-Aldrich, Burlington, MA, USA) was added to the solution at 0.1 wt% as a surfactant. Sample fluid including particles was injected via a syringe pump (LEGATO 111, KD Scientific Inc., Holliston, MA, USA), whose flow rate was controlled from 1 to 50 μL/min. *E. coli* BL21 competent cells were purchased from Thermo Fisher Scientific (USA). Before experiments, *E. coli* cells were cultured overnight in 2 mL Luria Broth (LB) medium (ampicillin added at 50 µg/mL) in an incubator at 37 °C with 200 rpm shaking. Then, 500 μL of cell culture was transferred to 1.5 mL of fresh growth media with 0.1 mM L-arabinose and cultured in an incubator at 37 °C with 200 rpm shaking. Before the experiment, the samples were centrifuged and washed twice with PBS.

The focusing positions of the particles were recorded and captured at 4 cm downstream from the inlet using a complementary metal-oxide semiconductor (CMOS) camera (Suzhou ZWO Co., Ltd., Suzhou, China) mounted on an optical microscope (BX-60, Olympus, Tokyo, Japan). For the precise analysis of the particle focusing width and position, bright-field and fluorescent images were captured from the top of the microchannel, utilizing exposure times of 50 ms and 800 ms. All the analyses and post-processing of the captured fluorescent images were carried out using the open-source ImageJ software 1.52a (NIH, New York, NY, USA), wherein the fluorescent intensity profiles in the microchannel were extracted and fitted with a Gaussian distribution. To compare the degree of particle focusing, the normalized fluorescence intensity was used as the intensity value, as in the previous study [17].

### 2.2. Principle of Particle Focusing and Enrichment

According to previous studies [17,18,19,20], depending on the diameter (*d*) of the particle, the particles inside viscoelastic fluids experience three kinds of forces; one is inertial lift force (*F_L_*), the other is elastic lift force (*F_E_*), and another is drag force (*F_D_*). The inertial lift force (*F_L_*) consists of the shear gradient lift force and the wall lift force. The shear gradient lift force drives the particles near the channel center toward the wall, whereas the wall lift force pushes the particles close to the wall toward the channel center. On the other hand, the elastic force (*F_E_*) originates from an imbalance in the distribution of the first normal stress (*N*_1_), which contributes to lateral particle migration. Therefore, particle migration in viscoelastic fluid results from both the inertial and viscoelastic effect, and the particle trajectories and equilibrium positions under fluid flow are determined by the competition of these forces [19]. When a particle moves through a fluid or the fluid flows past the particle, the viscous drag force (*F_D_*) appears. That is, it is due to the velocity difference between particle and fluid and it can also affect the particle migration. It can be expressed as the following equation.

The inertial lift force (*F_L_*), elastic force (*F_E_*), and viscous drag force (*F_D_*) in a uniform Stokes flow can be expressed as the following [2,21,22]:

(1) $$F_{L}\;=\;\frac{\rho_{f}{U_{max}}^{2}d^{4}}{{D_{h}}^{2}}f_{L}\left(Re,x_{c}\right)$$

(2) $$F_{E}\;=\;f_{E}d^{3}\nabla N_{1}=-2f_{E}d^{3}\eta_{p}\lambda \nabla {\dot{\gamma }}^{2}\;$$

(3) $$F_{D}\;=\;3\pi \mu_{f}d\left(v_{f}-v_{p}\right)$$

where *ρ_f_* is the fluid density, *U_max_* is the maximum velocity of the channel flow, *f_L_* (*Re*, *x_c_*) is the inertial lift coefficient, *d* is the diameter of particle, *Re* is the Reynolds number, *f_E_* is the elastic lift coefficient, *η_p_* is the polymeric contribution to the solution viscosity, *λ* is the relaxation time of the viscoelastic fluid, $\dot{\gamma }$ is the characteristic shear rate, *μ_f_* is the dynamic viscosity of the fluid, and *v_f_* and *v_p_* represent the velocities of fluid and particles, respectively.

The number of particle focusing points is determined by the Reynolds number (*Re* = *ρU_max_D_h_*/*μ*) and Weissenberg number (*Wi* = *λ*$\dot{\gamma }$ = 2*λU_max_*/*D_h_*), and the fluid elasticity (*El* = *Wi*/*Re* = 2*λμ*/(*ρD_h_*^2^)) is defined as the ratio of the Weissenberg number to the Reynolds number [2]. *Re* is used to quantify the relative importance of the inertial and viscous effect, and *Wi* is used to quantify the viscoelastic effects of a fluid and to describe the ratio of elastic and viscous forces. *El* means the relative importance of the elastic effect to the inertial effect. That is, it is possible to identify the major force responsible for the particle focusing phenomena in a viscoelastic fluid through *El.* The related values for *Re*, *Wi*, and *El* were calculated and presented in Tables S4 and S5.

The blockage ratio (*β* = *d*/*D_h_*) is also an important parameter that describes the tendency of particle focusing under viscoelastic fluid flow [23], and is defined as the ratio of the particle diameter (*d*) to the hydraulic diameter of the channel (*D_h_*). Particle migration and focusing under viscoelastic fluid flow are more distinct and tighter at a high blockage ratio (*β* > 0.07) due to the elastic force on the particle induced by normal stress difference (∇*N*_1_), which is directly proportional to the cube of the particle diameter (*d*), as shown in Equation (2) [23,24].

The elasto-inertial particle focusing in a viscoelastic fluid is affected by the inertial lift force and elastic force [18,19], with the cross-sectional shape of microchannel also playing a significant role in particle focusing and migration. In our previous work [14], it was shown that the impact of the reflex angles on elasto-inertial particle focusing was closely related with the distribution of shear rates within the channel as well as the fluid inertia. That is, the reflex angles in the cruciform geometry generated distinct regions of varying shear rates, with higher shear rates near the reflex corners and lower shear rates at the channel center. While randomly dispersed particles in the inlet reached certain equilibrium positions for the microchannel with square cross-section [25], they could be perfectly focused to the channel center of the microchannel with a cruciform cross-section.

## 3. Results and Discussion

To evaluate the degree of particle focusing and enrichment, we utilized the focusing efficiency and enrichment factor. The focusing efficiency was defined as the fraction of particles in the central region within 1/5 of the channel width (5 μm for square microchannel, 9 μm for cruciform microchannel), and the enrichment factor was defined as the ratio of particle fraction in the central outlet to particle fraction in the other four outlets. They were obtained from the normalized fluorescence intensity graph via Gaussian distribution [26]. The focusing efficiency and enrichment factor of submicron-sized particles were investigated by varying the flow rate, blockage ratio (*β*), and PEO concentration. We used the normalized fluorescence intensity to evaluate the focusing and enriching capability of a cruciform microchannel, which was compared with that of a square microchannel. Next, focusing and enrichment experiments for bacteria were carried out to verify the application of the device for biological submicron-sized samples. In this study, all experiments to obtain the data including focusing efficiency and enrichment factor were carried out three times (N = 3).

### 3.1. Submicron-Sized Particle Focusing and Enrichment in a Viscoelastic Fluid

In this work, the effects of blockage ratio (*β*) and concentration of PEO solution on the elasto-inertial focusing were investigated for two microchannels with different cross-sections; one had a square and the other had a cruciform cross-section. Two microchannels had the same hydraulic diameter (*D_h_*) of 25 μm and the same channel length of 40 mm. Under the combined influence of the driving forces (elastic lift force, wall effect lift force, etc.), the particles that were randomly distributed at the inlets of the microchannel laterally migrated toward the center of the channel after passing through the microchannel.

**Figure 2:**
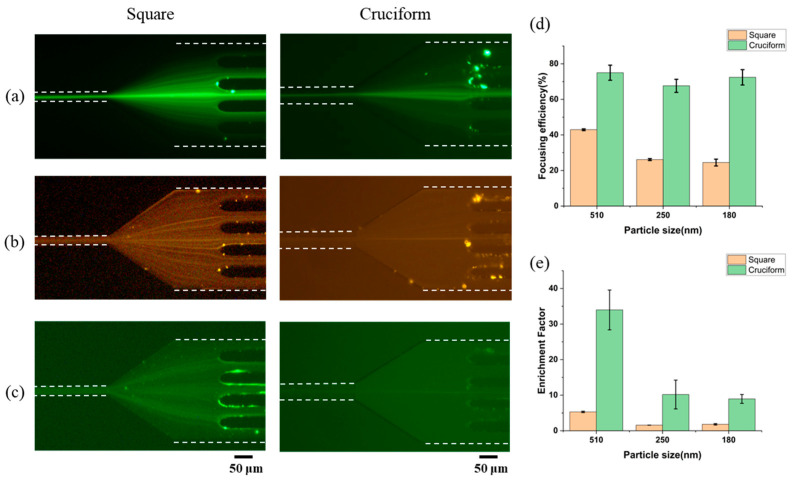
Fluorescence images of (**a**) 510 nm particle, (**b**) 250 nm particle, and (**c**) 180 nm particle in square and cruciform microchannels under viscoelastic fluid consisting of 0.1 wt% PEO aqueous solution (flow rate: 10 μL/min). (**d**) Focusing efficiencies and (**e**) enrichment factor plotted for three different particle sizes. The dotted lines indicate the ends of the microchannel walls, which were obtained from bright-field images under the same experimental conditions.

First, the effect of blockage ratio on particle focusing was investigated in viscoelastic fluid of 0.1 wt% PEO (M_w_ = ~2 MDa) solution whose flow rate was 10 μL/min. Figure 2a–c shows the fluorescent images of distributed particles at the end of a square microchannel and a cruciform microchannel, respectively. Three different-sized particles (510, 250, and 180 nm) with blockage ratios of 0.02, 0.01, and 0.007 were injected at flow rates ranging from 1 to 50 μL/min (Figures S1 and S2). As shown in Figures S1 and S2, 250 and 180 nm particles were barely focused on the center of the square channel. However, 250 nm particles were focused within a flow rate range of 1 to 10 μL/min and 180 nm particles within a flow rate range of 5 to 10 μL/min in the cruciform channel. These results confirm that particle focusing is greatly affected by the cross-sectional shape of the channel. Figure 2d,e shows the focusing efficiency and enrichment factor for both microchannels. At the flow rate of 10 μL/min, the 510 nm particles (*β* = 0.02) in the square microchannel were focused at the center and corners of the microchannel and showed a focusing efficiency of about 40% by the elasto-inertial particle focusing, which was the result of competition between the inertial lift force (*F_L_*) and elastic force (*F_E_*) in a viscoelastic fluid flowing [27]. Smaller particles (250 and 180 nm) showed focusing efficiencies below 30% due to their lower blockage ratios (*β* = 0.01, 0.007 < 0.07). On the other hand, the cruciform microchannel achieved focusing efficiencies above 70% for all particle sizes at the flow rate of 10 μL/min. That is, the submicron-sized particles could be focused to a single point at the center of the microchannel because they were affected by the four reflex angles of the microchannel as well as by elasto-inertial force in a viscoelastic fluid. However, in the previous study [14], particles with the blockage ratio above 0.02 (>2 μm) could be only focused. It was because the elastic force, which was proportional to the cube of the particle size, decreased slowly when compared to the inertial force that was proportional to the fourth power of the particle size. That is, the elastic force was still dominant due to four corners with reflex angles of 270°, so particles with lower blockage ratio could be focused even though the channel size decreased. The cruciform microchannel showed 1.8 times higher focusing efficiency than the square microchannel. However, 100 nm particles (*β* = 0.004) were not focused in both microchannels (Figure S3).

In the square microchannel, 510 nm particles showed an enrichment factor close to 5.3 ± 0.2, but the enrichment factor of the 250 and 180 nm particles did not exceed 2 due to the low focusing efficiency of the square microchannel. On the other hand, in the cruciform microchannel, 510 nm particles showed an enrichment factor of 34 ± 5.6, and 250 and 180 nm particles showed the enrichment factor of 10.2 ± 4.1 and 9.0 ± 1.2 due to the high focusing efficiency by the effect of the four reflex angles of the microchannel on the particles.

**Figure 3:**
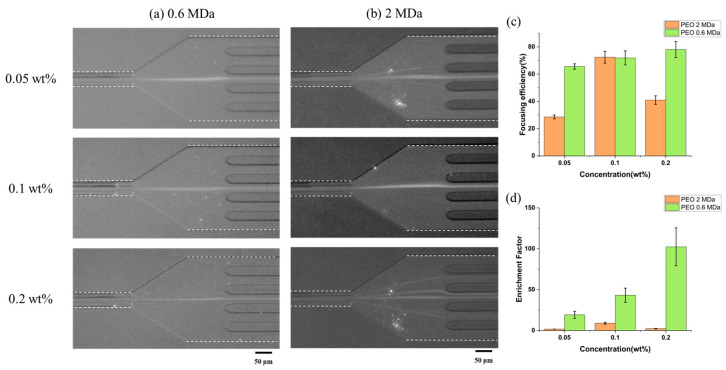
Fluorescence images in a cruciform microchannel for the concentration of PEO solution with the different molecular weights of (**a**) ~0.6 MDa and (**b**) ~2 MDa. (**c**) Focusing efficiency and (**d**) enrichment factor according to the concentration of PEO solution (particle size: 180 nm, flow rate: 10 μL/min).

Next, the effect of PEO concentration on the elasto-inertial focusing of submicron-sized particles was investigated in the cruciform microchannel. Because the cruciform microchannel showed the better performance for focusing and enriching submicron-sized particles than the square microchannel did, we just focused on the characterization for the cruciform microchannel. Figure 3 shows the fluorescence images and normalized intensities of 180 nm particles in a cruciform microchannel at the flow rate of 10 μL/min as functions of the concentration of PEO aqueous solution, which increased from 0.05 wt% to 0.1 wt% and 0.2 wt%. According to the results of a previous study [11], as the concentration of PEO solution increases, the elastic force also increases, which makes particles move toward the channel center. However, due to the shear shinning phenomenon, the number of particles moving to the edge of the channel rather than the channel center also increases. Therefore, the effective particle enrichment can be realized only for PEO solutions with a proper concentration under certain flow rate ranges. On the other hand, the shear thinning phenomenon did not occur even for 0.2 wt% PEO solution with low Mw of ~0.6 MDa in the cross-shaped channel, so we could obtain higher focusing efficiency and enrichment factor than others (Figure 3).

In these experiments, we also used two kinds of PEO solutions with different molecular weight (M_w_) of ~0.6 MDa and ~2.0 MDa. The focusing efficiencies for the PEO solution with high M_w_ increased from 2.86 ± 1.4% to 72.4 ± 4.3% and 41.0 ± 3.1%, while those for the PEO solution with low M_w_ increased from 65.6 ± 2.0% to 71.9 ± 5.2% and 78.1 ± 5.9%. On the other hand, the enrichment factors for the PEO solution with high M_w_ were 1.8 ± 0.0, 9.0 ± 1.2, and 2.3 ± 0.3, while those for the PEO solution with low M_w_ were 19.2 ± 4.5, 43.2 ± 8.7, and 102.4 ± 23.2, respectively. When focusing and enriching the submicron-sized particles, the PEO solution with low M_w_ had better performance than that with high M_w_. It is because the polymer solution with lower M_w_ possesses less-intensive shear-thinning behavior than that with higher M_w_ [11], and the shear-thinning effect associated with polymer solutions with higher M_w_ may also aggravate the focusing performance of the microchannel [28]. Therefore, it should be taken into account during the focusing and enriching experiments of the submicron/nano-sized particles.

### 3.2. Bacteria Focusing and Enrichment

**Figure 4:**
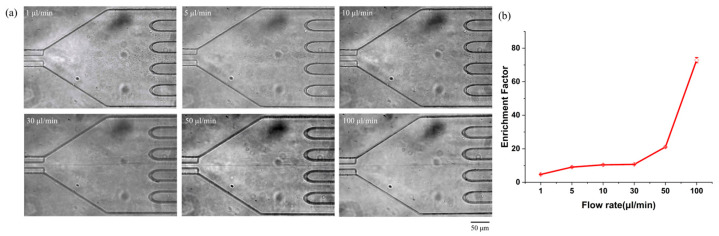
Bacteria (*Escherichia coli*) enriched using a microchannel with a cruciform cross-section in viscoelastic fluid consisting of 0.1 wt% PEO (M_w_ = ~2 MDa) in PBS solution according to the flow rates. (**a**) Optical microscopy images, (**b**) enrichment factor for bacteria.

To verify the application of the device for biological submicron-sized samples, enrichment experiments were conducted using *Escherichia coli* (*E. coli*) bacterial cells in the cruciform microchannel. The bacterial samples, including 1.8 × 10^6^ cells/mL, were vortexed prior to injection to prevent aggregation. It was possible to visualize the stream of bacterial flows by bright-field observation without fluorescent staining thanks to its average diameter of 900 nm. A 0.1 wt% PEO (M_w_ = ~2 MDa) in phosphate-buffered saline (PBS) solution was injected into the channel inlet for bacteria focusing and enrichment, whose flow rate was 1~100 µL/min. Figure 4a shows the optical microscopy image of bacterial cells, which was obtained using a CMOS camera set to a frame rate and exposure time of 30 ms (Video S1). Due to the sufficient elasto-inertial force of the cruciform microchannel, bacterial cells inside the microchannel were focused and directed to the center outlet among the five outlets. Figure 4b depicts the enrichment factor of bacteria at the outlet as the flow rate increases. The enrichment factor of bacteria was 4.7 ± 0.2 at a low flow rate of 1 µL/min and increased to 10.4 ± 0.2 at a flow rate of 10 µL/min, and the bacteria were almost focused to the channel center (focusing efficiency: 95%) and then moved out to the center outlet, showing an enrichment factor of 72.9 ± 1.6 at a flow rate of 100 µL/min.

In the case of submicron-sized particles (510 nm particles in Figure S2a), as the flow rate increased, the inertial force was more dominant compared to elastic force above the flow rate of 50 mL/min, which caused the particles to move away from the channel center. On the other hand, in the case of larger bacteria (average diameter of 900 nm), the inertial and elastic forces were comparable up to a flow rate of 100 mL/min, so the particles were still forced toward the channel center, which further increased the enrichment factor.

## 4. Conclusions

In this work, we proposed a novel microfluidic device with a hybrid microchannel with a square and cruciform cross-section to focus and enrich the submicron-sized particles and bacteria in PEO (polyethylene oxide) solution. The cruciform microchannel demonstrated a significant improvement in particle focusing on the center of the microchannel when compared to a square microchannel with an equivalent hydraulic diameter. This improvement can be attributed to the distinct geometry of the channel, which features four reflex angles. The four corners with 270° reflex angles enabled the cruciform cross-section to focus on up to 180 nm polystyrene particles without shear thinning. Furthermore, PEO solutions with low Mw performed better than those with high Mw due to less intensive shear thinning. The focusing and enrichment power of the cruciform microchannel also demonstrated biological particles such as bacteria. Therefore, the microfluidic device with a cruciform microchannel has great potential for focusing and enriching submicron particles, such as bacteria, and can be used in single-cell analysis and flow cytometry.

## Data Availability

No new data were created or analyzed in this study. Data sharing is not applicable to this article.

## Supplementary Materials

The following supporting information can be downloaded at: https://www.mdpi.com/article/10.3390/bios15060370/s1. Figure S1: Fluorescence images and normalized intensities inside channel and at outlet of square microchannel under viscoelastic fluid consisting of 0.1 wt% PEO (Mw = ~2 MDa) aqueous solution according to various flow rate. (a) 510 nm particle, (b) 250 nm particle, (c) 180 nm particle; Figure S2: Fluorescence images and normalized intensities inside channel and at outlet of cruciform microchannel under viscoelastic fluid consisting of 0.1 wt% PEO (M_w_ = ~2 MDa) aqueous solution according to various flow rate. (a) 510 nm particle, (b) 250 nm particle, (c) 180 nm particle; Figure S3: Fluorescence images and normalized intensities in square and cruciform microchannel under viscoelastic fluid consisting of 0.1 wt% PEO (Mw = ~2 MDa) aqueous solution (particle size: 100 nm, flow rate: 10 μL/min). (a) Square microchannel, (b) Cruciform microchannel; Table S1: (a) Pressure drop and (b) channel deformation in square and cruciform microchannel (Mw = 2 MDa); Table S2: (a) Pressure drop and (b)) channel deformation in square and cruciform microchannel (Mw = 0.6 MDa); Table S3: Rheological properties of PEO solutions with molecular weight (Mw) of 2 MDa and 0.6 MDa; Table S4: Dimensionless numbers in (a) square and (b) cruciform microchannel (Mw = 2 MDa); Table S5: Dimensionless numbers in (a) square and (b) cruciform microchannel (Mw = 0.6 MDa); Video S1: Bacteria focusing on the cruciform microchannel.
