# Supplementary material for: Improving the Enrichment of Submicron-Sized Particles by Size Decreasing of Cruciform Cross-Sectional Microchannel in Viscoelastic Microfluidics
Source: Biosensors (Basel). 2025 Jun 9;15(6):370. doi: 10.3390/bios15060370 (PMC12191125; doi:10.3390/bios15060370)
Supplement: Supplementary file 1 [file biosensors-15-00370-s001.zip › biosensors-3646844-supplementary.pdf]

Improving the Enrichment of Submicron-Sized Particles by Size Decreasing of Cruciform Cross-Sectional Microchannel in Viscoelastic Microfluidics

Jaekyeong Jang [1,†], Eunjin Kim [2,3,†], Sungdong Kim [1,4], Ok-Chan Jeong [3], Sangwook Lee [2,5,*] and Younghak Cho [1,4,*]

[1] Department of Mechanical Design and Robot Engineering, Seoul National University of Science and Technology, Seoul 01811, Republic of Korea; jjangy5720@naver.com (J.J.); sdkim@seoultech.ac.kr (S.K.)
[2] PCL Inc., Seoul 08510, Republic of Korea; kitty97@oasis.inje.ac.kr
[3] Department of Biomedical Engineering, Inje University, Gimhae-si 50834, Republic of Korea; memsoku@inje.ac.kr
[4] Department of Mechanical System Design Engineering, Seoul National University of Science and Technology, Seoul 01811, Republic of Korea
[5] mCureX, Seoul 05854, Republic of Korea
* Correspondence: swlee@pclchip.com (S.L.); yhcho@seoultech.ac.kr (Y.C.); Tel.: +82-2-2144-3901 (S.L.); +82-2-970-6361 (Y.C.)
† These authors contributed equally to this work.

Supplementary Information

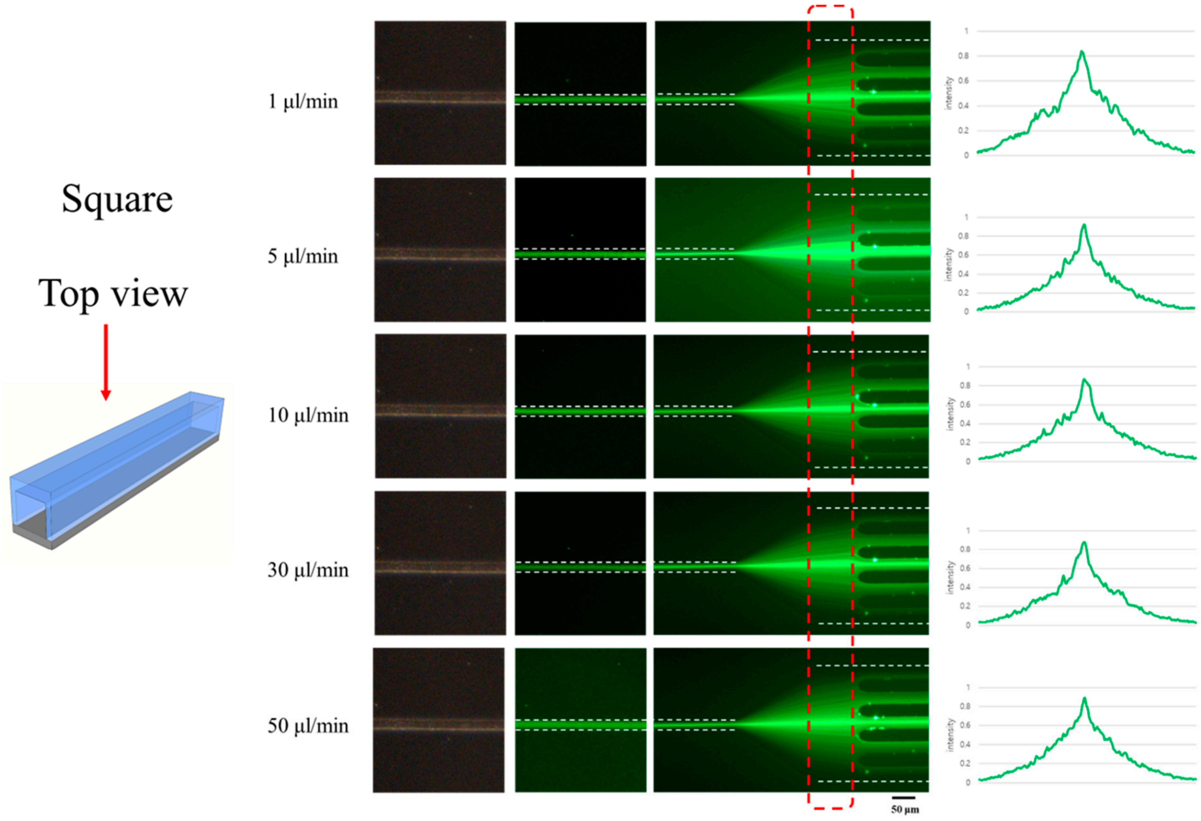

(a) 510 nm particle

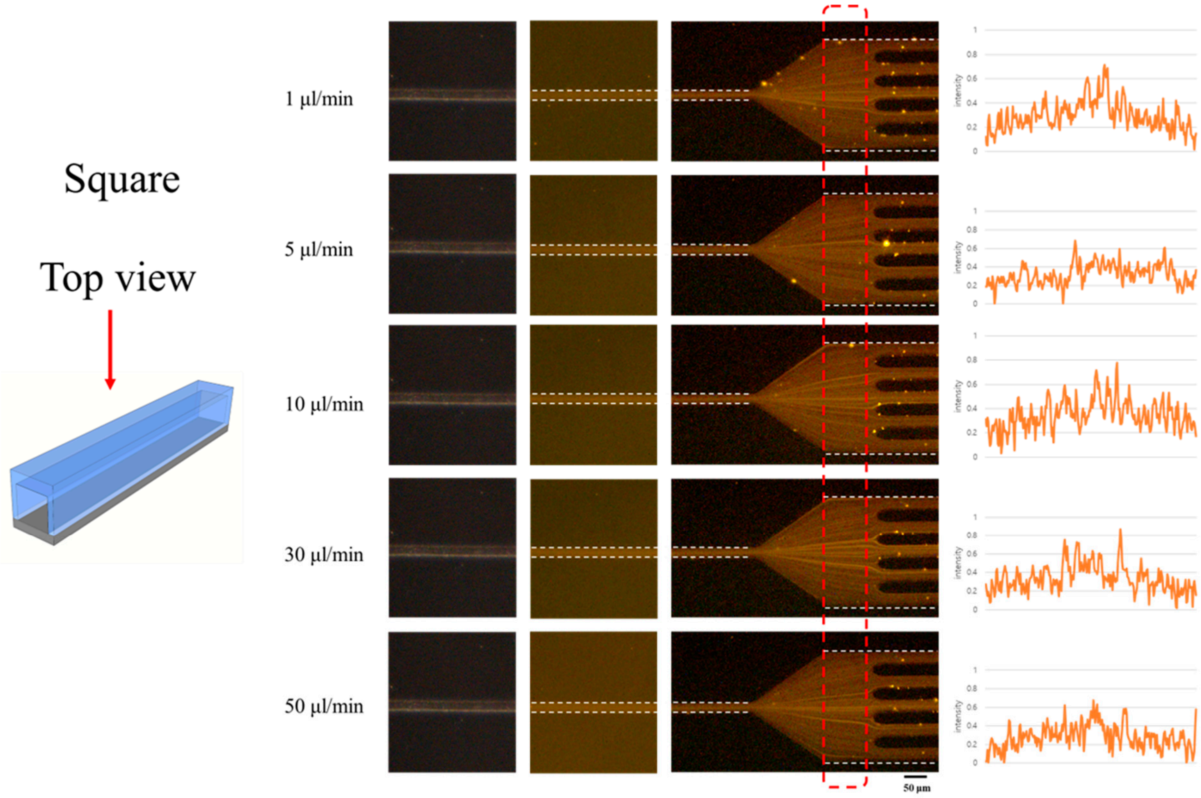

(b) 250 nm particle

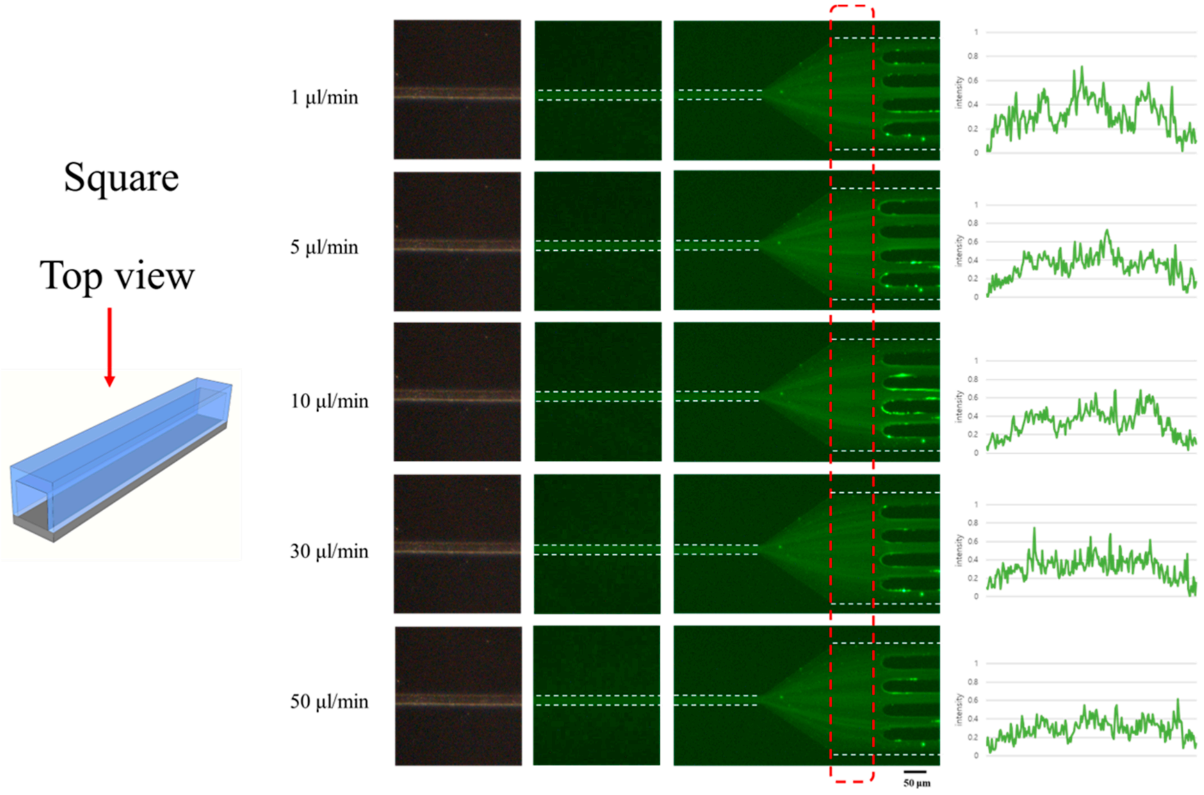

(c) 180 nm particle

Figure S1. Fluorescence images and normalized intensities inside channel and at outlet of square microchannel under viscoelastic fluid consisting of 0.1 wt% PEO (M_w = ~2 MDa) aqueous solution according to various flow rate. (a) 510 nm particle, (b) 250 nm particle, (c) 180 nm particle.

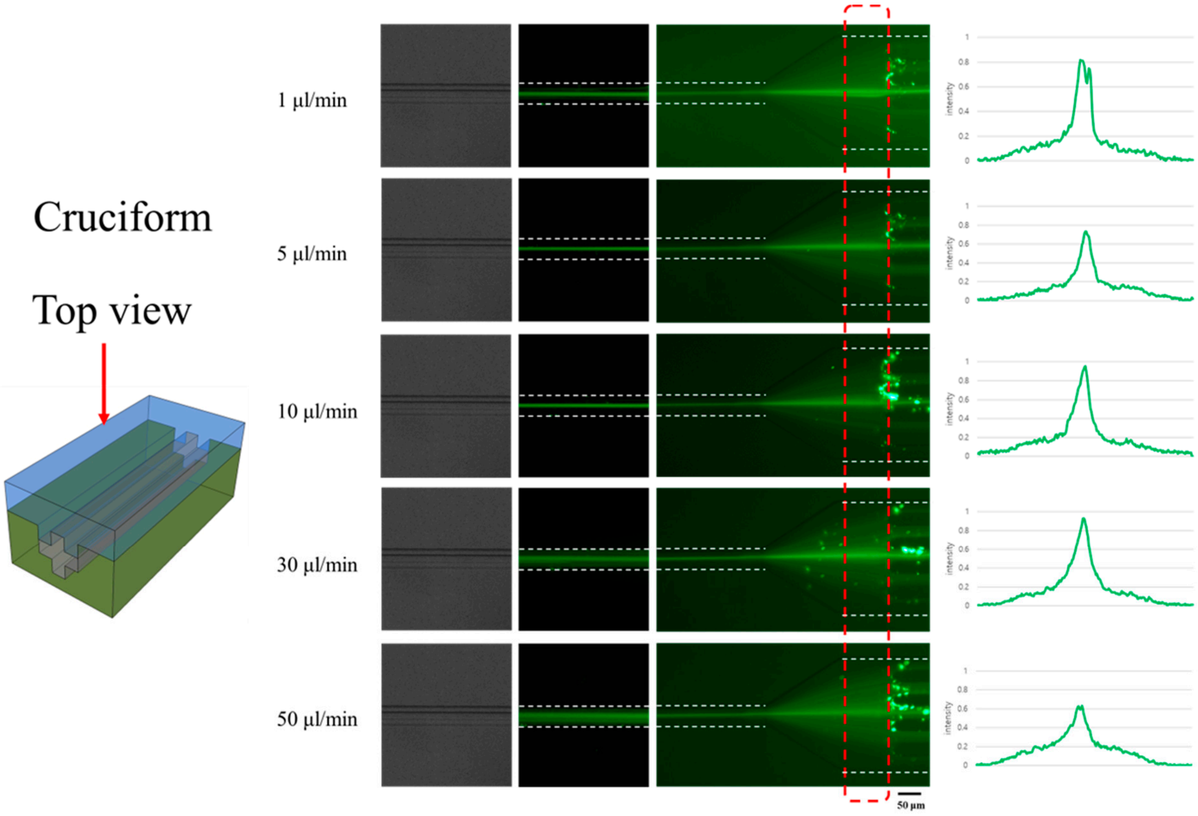

(a) 510 nm particle

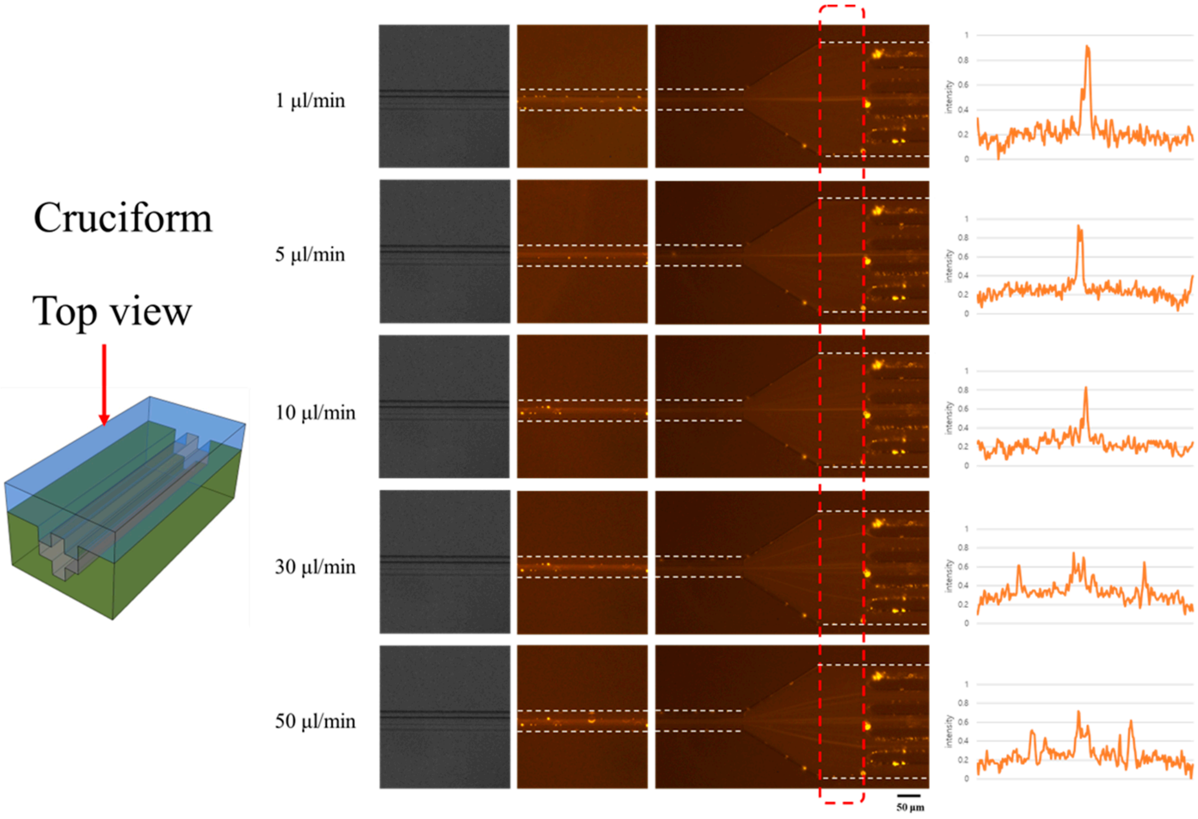

(b) 250 nm particle

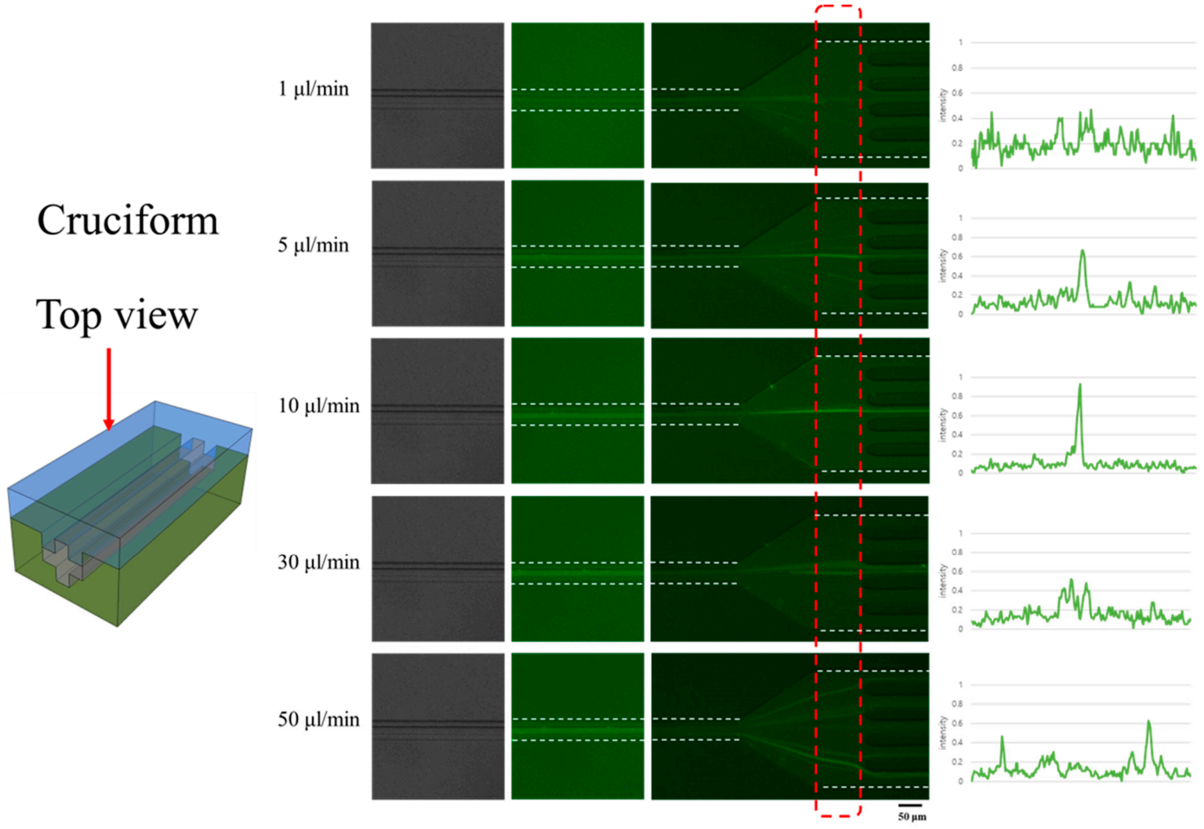

(c) 180 nm particle

Figure S2. Fluorescence images and normalized intensities inside channel and at outlet of cruciform microchannel under viscoelastic fluid consisting of 0.1 wt% PEO (M_w = ~2 MDa) aqueous solution according to various flow rate. (a) 510 nm particle, (b) 250 nm particle, (c) 180 nm particle.

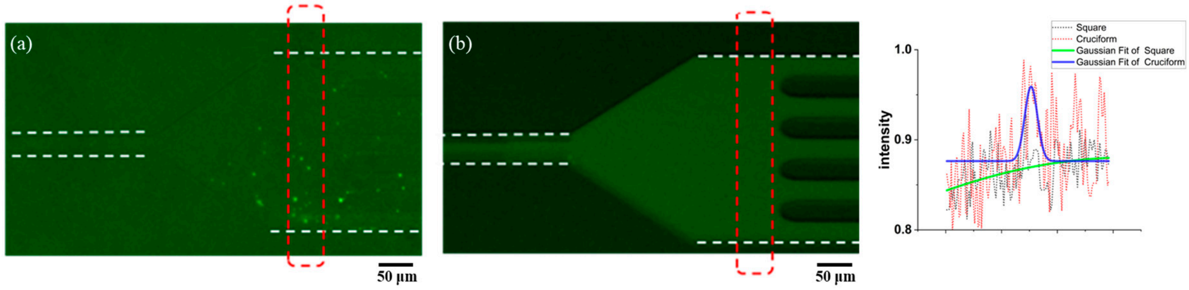

Figure S3. Fluorescence images and normalized intensities in square and cruciform microchannel under viscoelastic fluid consisting of 0.1 wt% PEO (M_w = ~2 MDa) aqueous solution (particle size: 100 nm, flow rate: 10 μL/min). (a) Square microchannel, (b) Cruciform microchannel.

Table S1. (a) Pressure drop and (b) channel deformation in square and cruciform microchannel (M_w = 2 MDa).

(a) Pressure drop [16]

**Q (μL/min)**	**Square microchannel**			**Cruciform microchannel**		
	**PEO Concentration (wt%)**			**PEO Concentration (wt%)**		
	**0.05**	**0.1**	**0.2**	**0.05**	**0.1**	**0.2**
	**ΔP(kPa)**	**ΔP(kPa)**	**ΔP(kPa)**	**ΔP(kPa)**	**ΔP(kPa)**	**ΔP(kPa)**
1	68.80	86.84	130.53	38.23	48.24	72.51
5	344.00	434.18	652.63	191.15	241.21	362.57
10	688.13	868.35	1305.26	382.29	482.42	725.14
30	2064.38	2605.06	3915.78	1146.88	1447.25	2175.43
50	3440.64	4341.76	6526.29	1911.47	2412.09	3625.72
100	6881.28	8683.52	13052.59	3822.93	4824.18	7251.44

(b) Channel deformation

**Q (μL/min)**	**Square microchannel**			**Cruciform microchannel**		
	**PEO Concentration (wt%)**			**PEO Concentration (wt%)**		
	**0.05**	**0.1**	**0.2**	**0.05**	**0.1**	**0.2**
	**Δd(μm)**	**Δd(μm)**	**Δd(μm)**	**Δd(μm)**	**Δd(μm)**	**Δd(μm)**
1	0.005	0.007	0.010	0.010	0.012	0.018
5	0.027	0.034	0.051	0.048	0.061	0.092
10	0.054	0.068	0.102	0.097	0.122	0.184
30	0.161	0.204	0.306	0.290	0.366	0.551
50	0.269	0.339	0.510	0.484	0.611	0.918
100	0.538	0.678	1.020	0.968	1.221	1.836

[16] Hsieh S, Lin C, Huang C, Tsai H (20004) Liquid flow in a micro-channel. J Micromech Microeng 14:436–445.

Table S2 (a) Pressure drop and (b)) channel deformation in square and cruciform microchannel (M_w = 0.6 MDa).

(a) Pressure drop [16]

Q (µL/min)	Square microchannel			Cruciform microchannel		
	PEO Concentration (wt%)			PEO Concentration (wt%)		
	0.05	0.1	0.2	0.05	0.1	0.2
	ΔP(kPa)	ΔP(kPa)	ΔP(kPa)	ΔP(kPa)	ΔP(kPa)	ΔP(kPa)
1	56.57	63.30	85.47	31.43	35.16	47.48
5	282.87	316.48	427.35	157.15	175.82	237.42
10	565.75	632.97	854.71	314.30	351.65	474.84
30	1697.25	1898.91	2564.12	942.91	1054.95	1424.51
50	2828.74	3164.85	4273.54	1571.52	1758.25	2374.19
100	5657.48	6329.69	8547.07	3143.05	3516.49	4748.38

(b) Channel deformation

Q (µL/min)	Square microchannel			Cruciform microchannel		
	PEO Concentration (wt%)			PEO Concentration (wt%)		
	0.05	0.1	0.2	0.05	0.1	0.2
	Δd(µm)	Δd(µm)	Δd(µm)	Δd(µm)	Δd(µm)	Δd(µm)
1	0.004	0.005	0.007	0.008	0.009	0.012
5	0.022	0.025	0.033	0.040	0.045	0.060
10	0.044	0.049	0.067	0.080	0.089	0.120
30	0.133	0.148	0.200	0.239	0.267	0.361
50	0.221	0.247	0.334	0.398	0.445	0.601
100	0.442	0.495	0.668	0.796	0.890	1.202

Table S3 Rheological properties of PEO solutions with molecular weight (M_w) of 2 MDa and 0.6 MDa [11].

	M_w = 2 MDa			M_w = 0.6 MDa		
	PEO Concentration (wt%)			PEO Concentration (wt%)		
	0.05	0.1	0.2	0.05	0.1	0.2
Density (kg/m^3)	996	996	996	996	996	996
Zero-shear viscosity μ_0 (mPa·s)	1.26	1.59	2.39	1.04	1.16	1.56
Effective relaxation time λ_e (ms)	4.31	6.76	10.61	0.48	0.8	1.22
Overlap concentration c* (ppm)	858	858	858	1877	1877	1877

[11] Liu C, Ding B, Xue C, et al (2016) Sheathless focusing and separation of diverse nanoparticles in viscoelastic solutions with minimized shear thinning. Anal Chem 88:12547–12553.

Table S4 Dimensionless numbers in (a) square and (b) cruciform microchannel (M_w = 2 MDa).

(a) Square microchannel

Q (μL/min)	PEO Concentration (wt%)								
	0.05			0.1			0.2		
	Re	Wi	El	Re	Wi	El	Re	Wi	El
1	0.53	9.19	17.45	0.42	14.42	34.53	0.28	22.63	81.47
5	2.63	45.97	17.45	2.09	72.11	34.53	1.39	113.17	81.47
10	5.27	91.95	17.45	4.18	144.21	34.53	2.78	226.35	81.47
30	15.81	275.84	17.45	12.53	432.64	34.53	8.33	679.04	81.47
50	26.35	459.73	17.45	20.88	721.07	34.53	13.89	1131.73	81.47
100	52.70	919.47	17.45	41.76	1442.13	34.53	27.78	2263.47	81.47

(b) Cruciform microchannel

Q (μL/min)	PEO Concentration (wt%)								
	0.05			0.1			0.2		
	Re	Wi	El	Re	Wi	El	Re	Wi	El
1	0.29	5.11	17.45	0.23	8.01	34.53	0.15	12.57	81.47
5	1.46	25.54	17.45	1.16	40.06	34.53	0.77	62.87	81.47
10	2.93	51.08	17.45	2.32	80.12	34.53	1.54	125.75	81.47
30	8.78	153.24	17.45	6.96	240.36	34.53	4.63	377.24	81.47
50	14.64	255.41	17.45	11.60	400.59	34.53	7.72	628.74	81.47
100	29.28	510.81	17.45	23.20	801.19	34.53	15.43	1257.48	81.47

Table S5 Dimensionless numbers in (a) square and (b) cruciform microchannel (M_w = 0.6 MDa).

(a) Square microchannel

Q (μL/min)	PEO Concentration (wt%)								
	0.05			0.1			0.2		
	Re	Wi	El	Re	Wi	El	Re	Wi	El
1	0.64	1.03	1.62	0.57	1.70	2.98	0.43	2.60	6.10
5	3.18	5.16	1.62	2.86	8.52	2.98	2.14	13.01	6.10
10	6.36	10.32	1.62	5.72	17.05	2.98	4.27	26.03	6.10
30	19.08	30.96	1.62	17.16	51.14	2.98	12.81	78.08	6.10
50	31.80	51.61	1.62	28.60	85.23	2.98	21.35	130.13	6.10
100	63.60	103.21	1.62	57.19	170.45	2.98	42.70	260.27	6.10

(b) Cruciform microchannel

Q (μL/min)	PEO Concentration (wt%)								
	0.05			0.1			0.2		
	Re	Wi	El	Re	Wi	El	Re	Wi	El
1	0.35	0.57	1.62	0.32	0.95	2.98	0.24	1.45	6.10
5	1.77	2.87	1.62	1.59	4.73	2.98	1.19	7.23	6.10
10	3.53	5.73	1.62	3.18	9.47	2.98	2.37	14.46	6.10
30	10.60	17.20	1.62	9.53	28.41	2.98	7.12	43.38	6.10
50	17.67	28.67	1.62	15.89	47.35	2.98	11.86	72.30	6.10
100	35.33	57.34	1.62	31.77	94.70	2.98	23.72	144.59	6.10
